# Gα12 signaling regulates transcriptional and phenotypic responses that promote glioblastoma tumor invasion

**DOI:** 10.1038/s41598-023-49164-4

**Published:** 2023-12-16

**Authors:** Olga Meiri Chaim, Shunichiro Miki, Briana C. Prager, Jianhui Ma, Anthony Y. Jeong, Jacqueline Lara, Nancy K. Tran, Jeffrey M. Smith, Jeremy N. Rich, J. Silvio Gutkind, Shigeki Miyamoto, Frank B. Furnari, Joan Heller Brown

**Affiliations:** 1 Department of Pharmacology, University of California San Diego, Biomedical Sciences Building, 9500 Gilman Drive #0636, La Jolla, CA 92093-0636 USA; 2 Department of Cell Biology, Federal University of Paraná, Curitiba, Brazil; 3 Department of Medicine, University of California San Diego, La Jolla, CA USA; 4 Ludwig Institute for Cancer Research, San Diego Branch, La Jolla, CA USA; 5 Cleveland Clinic Lerner College of Medicine, Cleveland Clinic, Cleveland, OH USA; 6 Moores Cancer Center, University of California at San Diego, La Jolla, CA USA; 7 UPMC Hillman Cancer Center, Pittsburgh, PA USA; 8 Department of Neurology, University of Pittsburgh School of Medicine, Pittsburgh, PA USA

**Keywords:** Lipid signalling, RHO signalling, Cancer models, Cancer stem cells, Head and neck cancer, Oncogenes

## Abstract

In silico interrogation of glioblastoma (GBM) in The Cancer Genome Atlas (TCGA) revealed upregulation of *GNA12* (Gα12), encoding the alpha subunit of the heterotrimeric G-protein G12, concomitant with overexpression of multiple G-protein coupled receptors (GPCRs) that signal through Gα12. Glioma stem cell lines from patient-derived xenografts also showed elevated levels of Gα12. Knockdown (KD) of Gα12 was carried out in two different human GBM stem cell (GSC) lines. Tumors generated in vivo by orthotopic injection of Gα12KD GSC cells showed reduced invasiveness, without apparent changes in tumor size or survival relative to control GSC tumor-bearing mice. Transcriptional profiling of GSC-23 cell tumors revealed significant differences between WT and Gα12KD tumors including reduced expression of genes associated with the extracellular matrix, as well as decreased expression of stem cell genes and increased expression of several proneural genes. Thrombospondin-1 (*THBS1*), one of the genes most repressed by Gα12 knockdown, was shown to be required for Gα12-mediated cell migration in vitro and for in vivo tumor invasion. Chemogenetic activation of GSC-23 cells harboring a Gα12-coupled DREADD also increased THBS1 expression and in vitro invasion. Collectively, our findings implicate Gα12 signaling in regulation of transcriptional reprogramming that promotes invasiveness, highlighting this as a potential signaling node for therapeutic intervention.

## Introduction

G protein coupled receptors (GPCRs) transduce their signals through coupling to heterotrimeric G-proteins. Upon stimulation of GPCRs by binding of their cognate ligands, the G-protein alpha subunit (G_α_) exchanges GDP for GTP and in this activated state can bind to and regulate its downstream effectors. The effectors of the earliest discovered G-proteins of the G_s_, G_i_, and G_q_ families are the enzymes adenylate cyclase and phospholipase C. The last discovered G-protein family (G_12/13_), encoded by *GNA12* and *GNA13*, do not couple to these enzymes. Instead, the effectors for Gα12/Gα13 are GTP exchange factors (GEFs) for the low molecular weight GTPase RhoA^1,2^. Activation of Gα12 increases signaling to RhoA which elicits cytoskeletal responses^3–5^. More recently established mediators of RhoA signaling are the transcriptional co-activators YAP and MRTF-A^6,7^, which we previously linked to GSC and GBM tumor growth^8^.

The GPCRs that are most prominently coupled to G12/13 and RhoA signaling include those for thrombin and the lysophospholipids LPA and S1P^9,10^. Our previous work and that of others demonstrated that activated GPCRs that couple to Gα12 and RhoA are efficacious mitogens and transcriptional activators^11–13^. These receptors are highly expressed in the brain, activated by ligands that are generated in response to inflammation and have been implicated in tumor growth and progression^14–16^. Levels of mRNA for GPCRs, such as PAR1 (the receptor for thrombin) and S1P2 and S1P3 (receptors for sphingosine 1-phosphate), are elevated in GBM^14,17,18^. In addition to enhanced expression of a wide range of GPCRs and increased availability of their ligands, Gα12 mRNA is particularly elevated in higher grades of glioma^8^. While multiple distinct Gα12-coupled GPCRs could regulate GBM progression in vivo, these receptors all ultimately converge on Gα12 to transduce their signals. Accordingly, we reasoned that knocking down Gα12, the nodal mediator of signaling through these GPCRs, would demonstrate the importance of this pathway and provide clues as to what signals contribute to its role in aggressiveness and progression of GBM tumors.

Here we examine in vitro and in vivo the properties of GSCs in which Gα12 expression is knocked down with shRNA. Loss of the Gα12 pathway diminished invasion of GBM tumor cells in vivo, in association with altered extracellular matrix, proneural and stem cell gene expression. Amongst the ECM components, thrombospondin-1 (*THBS1*), previously associated with tumor progression, was one of the most significantly downregulated in cells and tumors lacking Gα12. Our study highlights the importance of Gα12 as an integrator of signals from GPCRs to transcriptional responses that alter glioma cell phenotype and tumor invasion.

## Materials and methods

### Glioblastoma stem Cells

GSC23 and HK281 were provided by Dr. Frederick Lang, MD Anderson and Dr. Harley Kornblum, UCLA, respectively. GSC neurospheres were cultured in suspension flasks containing DMEM/F12 medium supplemented with B27 (Gibco) in a 5% CO_2_ 37 °C incubator. Lentivirus infection to knock down *GNA12* or *THBS1* or express Gα12 DREADD in GSC cells was followed by 48–96-h puromycin selection.

### Extreme limiting dilution assay

Neurospheres dissociated into cell suspension by Accutase treatment were counted and plated in fresh media in pentaplicate into 96-wells at 1–100 cells/well. After 21 days spheres were counted to estimate stem cell frequency by ELDA (http://bioinf.wehi.edu.au/software/elda/) using χ2 to determine pair-wise differences.

### Orthotopic GSC injections

1.5 or 5 × 10^5^ control or knocked down GSC23 cells tagged with near infrared IRFP720 were intracranially injected into the mouse brain (6 mice per group), using a stereotactic system as previously described^8^. Survival experiments were performed twice. Tumor size was estimated by fluorescence emission detection by FMT 2500 Fluorescence Tomography (Perkin Elmer) at 720 nm. The onset of neurologic sequelae in the control group was used to determine time of euthanasia. Mice were euthanized by CO2 inhalation in accordance with our institutional guidelines for animal welfare and experimental conduct at University of California at San Diego. Brain samples were collected, and tissue samples were processed for histological examination by H&E and anti-human nuclei IHC at UCSD CALM and MCC Biorepository and Tissue Technology Core.

### RNA analysis

Total RNA was isolated using Trizol reagent according to the manufacturer’s protocol, followed by RT-qPCR for relative quantification. RNA sequencing of tumors from mice injected with Gα12 KD or control GSC23 cells (4 mice tumors for biological replicates in each group) were submitted to RNA integrity analysis (Agilent Bioanalyzer, Tapestation results eRIN > 8.5), ribodepleted library preparation, and sequencing using Illumina NovaSeq 6000 (run set PE100 and 25 M reads). Gene-set enrichment analysis was performed using GSEA software.

### Migration/invasion assay

Uncoated or Geltrex-coated membranes of Transwell 24-well plates (8 µm pore size, Corning, Cat#3422) were used to assess migration and invasion, respectively, as detailed in Supplementary Material.

### Statistics

Statistical differences were analyzed using Graphpad Prism software version 8. Analysis of variance (ANOVA) followed by Tukey’s multiple test was applied for groups with several features. One-way ANOVA was used to analyze data from experiments with one independent variable, and two-way ANOVA for two independent factors. Data are presented as mean ± SEM and significances based on calculated probability values (*p < 0.05; **p < 0.01).

### Ethical approval

In vivo experiments were executed under approval of animal protocol by University of California San Diego Institutional Animal Care and Use Committee (IACUC) Office #S00192M, which were performed following the protocols in complying with federal regulations by USDA, APHIS, CFR, Title 9, Parts 1, 2, and 3. The study was reported in accordance with the recommendations of the ARRIVE guidelines.

## Results

### GBMs overexpress GNA12 and G⍺12—coupled GPCRs

**Figure 1:**
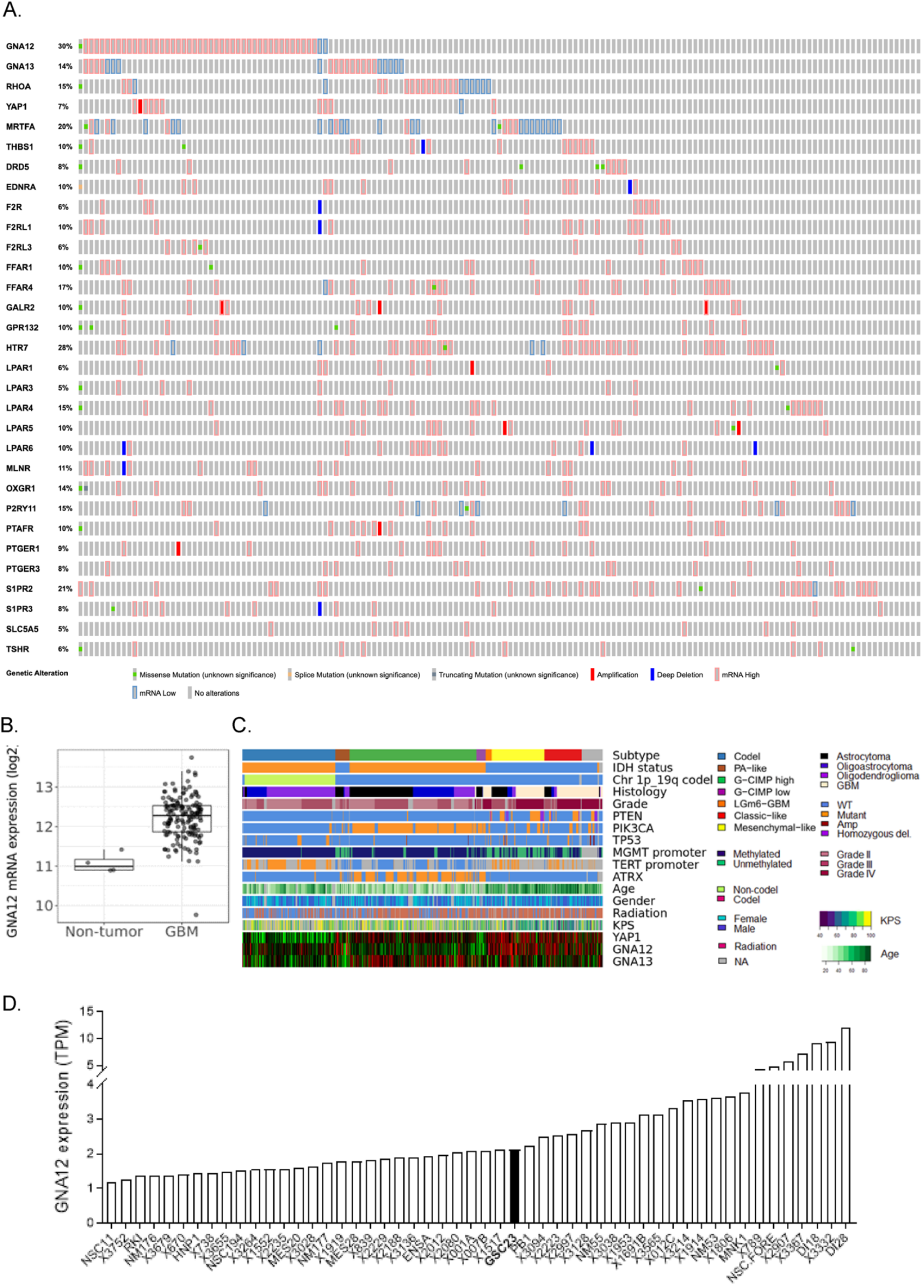
G⍺12 is upregulated in high grade glioblastoma tumor samples and GSC cell lines. (**A**) cBio oncoprint from TCGA PanCancer GBM study. Genomic alterations in *GNA12* and predicted G⍺12-coupled GPCRs. Z-scores relative to diploid samples RNA-Seq V2 RSEM: ± 1.5 threshold. (**B**) G⍺12 upregulation in GBM. RNA-Seq sample counts from Gliovis, *p* < 0.001. (**C**) Heatmaps of RNA-seq, whole exome, and additional clinical phenotype data aggregated from TCGA. (**D**) G⍺12 mRNA expression relative to neural stem cells in GSC cell lines.

We interrogated the TCGA database generated from patient GBM surgical specimens and determined that *GNA12* mRNA expression was elevated in 30% of 160 GBM patient samples profiled in TCGA, while its homolog, G⍺13, was far less frequently overexpressed (Fig. 1A). We also interrogated the TCGA for a series of GPCRs recently established to couple efficiently to G⍺12 (Supplemental Fig. 1). GPCRs that couple to G⍺12 and are altered in ≥ 5% of patients are shown in Fig. 1A. Notably, most GPCRs were overexpressed in fewer patients and in subsets of patients distinct from those with elevated *GNA12*. Overall expression of *GNA12* mRNA in GBM based on TCGA analysis by Gliovis was 2.2-fold higher than in normal brain (Fig. 1B).

We examined expression of *GNA12* in relation to the molecular classification and phenotypic characteristics of glioma samples, including consideration of their IDH1/p53/*PTEN* mutational status, tumor grade, patient age, and survival. GBMs have been also classified by transcriptional signatures into proneural, classical, and mesenchymal subtypes^19^. *GNA12*, but not *GNA13*, was highly expressed in the classical and mesenchymal GBM subtypes and enriched in elderly patients and those with worst performance status (Fig. 1C). Expression clustered with *YAP1*, a downstream transcriptional co-activator regulated through G⍺12-RhoA signaling^7,20,21^.

RNA-seq data from 40 patient-derived GSCs generated by the Rich laboratory and compiled and stored in a data base described previously^22,23^ was also analyzed; all GSCs were found to have levels of *GNA12* expression at least one SD above that of neural stem cells (Fig. 1D). The GSC23 cell line, established from a patient-derived xenograft of a recurrent and aggressive tumor^24^, and also used in our previous work^8^, was intermediate in its expression of *GNA12*, providing a representative model to examine the role of G⍺12 signaling in GBM growth.

GSCs were transduced by lentiviral-directed short hairpin RNAs (shRNAs) encoding either a control sequence not found in the mammalian genome or one of two non-overlapping G⍺12 sequences. G⍺12 mRNA levels were reduced by over 75% relative to control cells without significant compensatory changes in G⍺13 (Supplemental Fig. 2). Westerns on whole cell lysates also demonstrated an approximately 50% decrease in G⍺12 protein in the knockdowns compared to control cells (Supplemental Fig. 2).

**Figure 2:**
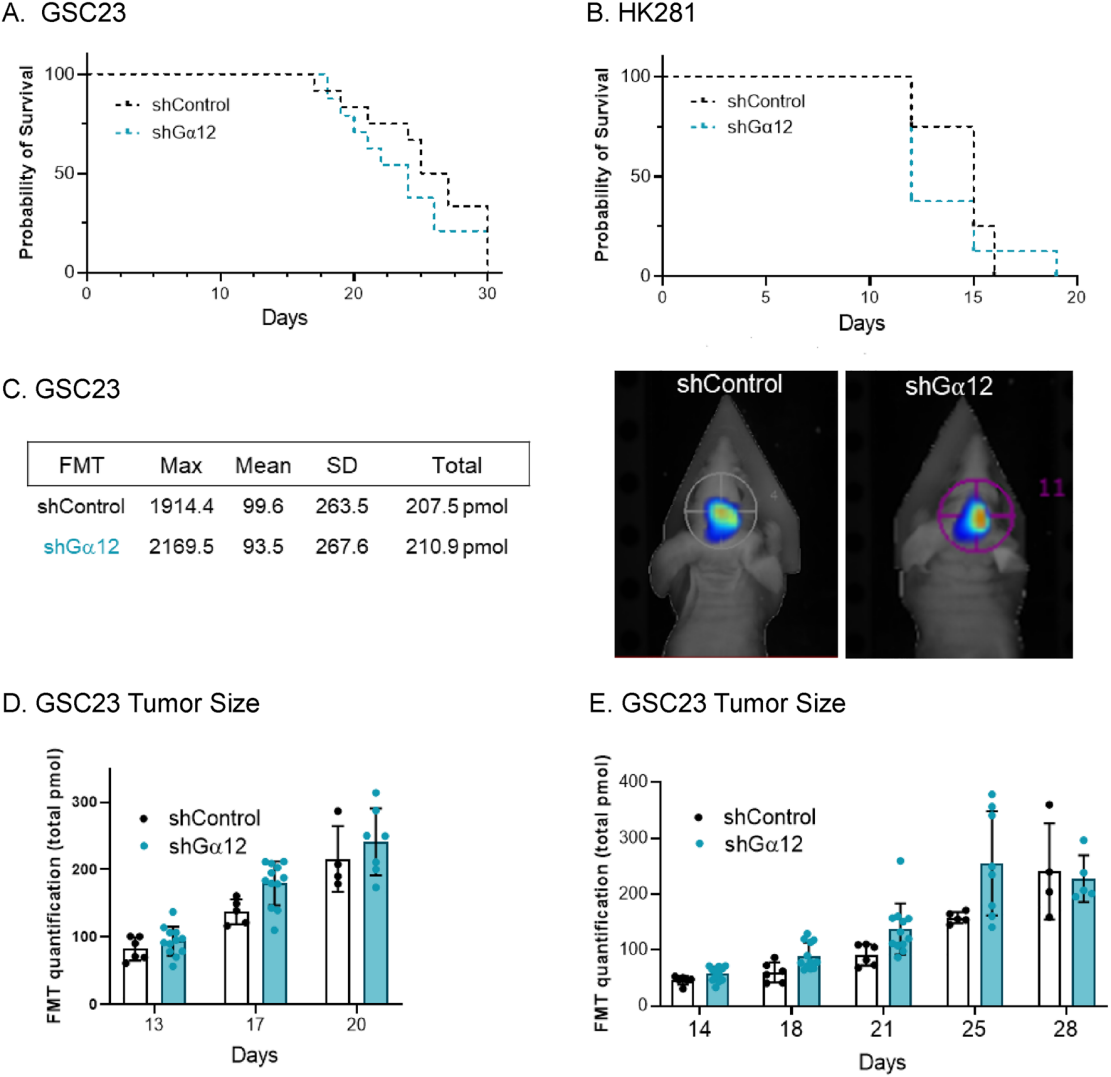
G⍺12 knockdown does not alter mouse survival or tumor size observed following orthotopic intracranial injection of GSC tumor cells. shRNA control or shG⍺12 knockdown GSC23 and HK281 cells labeled with IRFP720 were intracranially injected into syngeneic nu/nu mice. (**A**, **B**) Kaplan–Meier survival curves for GSC23 and HK281 control and shG⍺12 KD tumor-bearing mice (6 animals per group, in 2 independent experiments for GSC23, and 4 animals per group for HK281 in one experiment). Survival curves were not significantly different as determined using the Log rank test (**C**, **D**) Brain tumor growth of GSC23-engrafted mice as monitored using Fluorescence Molecular Tomography (FMT) emission at 720 nm. Representative FMT scan images at 17 days post intracranial injection and relative fluorescence quantification. (**E**) Additional set of animals injected with approximately 3 × 10^5^ GSC23 cells and analyzed for tumor growth monitored by FMT did not show significant differences in tumor size (*p* = 0.1; n = 6).

### Depletion of G⍺12 does not affect GBM lethality or tumor size

To determine whether G⍺12 protein signals are critical for tumorigenesis in the brain microenvironment we intracranially implanted mice with GSC23 control or G⍺12 shRNA transduced cells labeled with IRFP720. The experiment was repeated twice with 6 animals per group in each experiment. Inhibition of G⍺12 did not change overall survival of the tumor-bearing mice over a period of approximately 30 days (Fig. 2A). We used an additional GBM patient derived glioma stem cell, HK281, which our previous work established to show elevated G⍺12 mRNA (to an extent approximately double that of GSC23 cells)^8^. Knockdown of G⍺12 in HK281 was highly effective and occurred without concomitant changes in G⍺13 expression (Supplemental Fig. 2). Survival of mice implanted with G⍺12 KD HK 281 cells was not altered (Fig. 2B).

Although alterations in overall survival were not observed when G⍺12 expression was inhibited, we carried out additional analysis on GSC-23 cell implanted mice. Tumor size was assessed using fluorescent molecular tomography (FMT) of IRFP720-expressing WT and G⍺12 knockdown tumors as shown in Fig. 2C. Tumor size, assessed longitudinally, did not differ significantly between the mice bearing control and G⍺12 knockdown cells (Fig. 2D). In an additional series of orthotopic injections, we engrafted approximately half the number of GSC23 cells to minimize potential deleterious effects of massive tumor development and associated lethality. Here, again, there was no difference in the survival of the two groups of tumor-bearing mice followed until the time of sacrifice, and serial FMT imaging of tumor-bearing brains revealed insignificant fluorescence range distributions between groups (Fig. 2E). We also confirmed that G⍺12 mRNA levels remained downregulated in the KD tumors (Supplemental Fig. 2A,B).

### GNA12 is essential for in vivo tumor invasion

**Figure 3:**
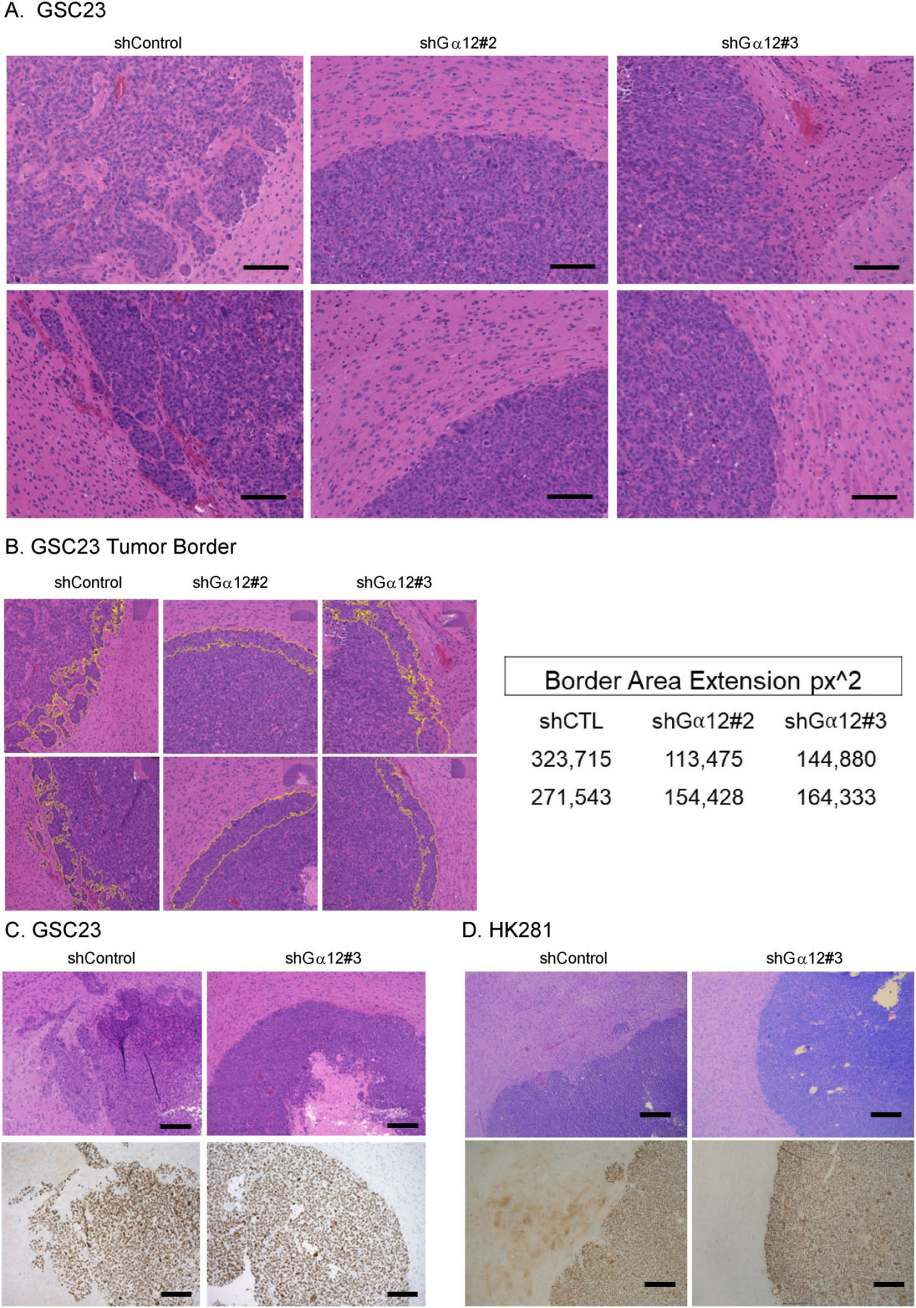
Orthotopic xenograft GSC tumors with G⍺12 knockdown exhibit diminished invasiveness. (**A**, **B**) Mouse brain cross sections at 10 days post GSC23 intracranial injection stained with H&E (hematoxylin and eosin) (2 per group). (**B**) Yellow lines define tumor border analyzed by digital pathology detection software QuPath for border area by square pixel of cross-sections (*p* < 0.05 vs. shControl, 2 per group) (**C**, **D**) H&E and corresponding IHC sections marking human cell nuclei (HPM, × 40) in tumors from GSC23 and HK281 tumor-bearing mice.

Tumor-bearing brains were harvested, sectioned, and stained with hematoxylin and eosin (H&E) or analyzed by immunohistochemistry (IHC) (Fig. 3A–C). GSC23 control cells generated tumors with typical irregular invasive GBM borders (Fig. 3A). In contrast, the tumor mass in mice injected with G⍺12 KD GSC23 cells was largely confined to the injection site and the tumor border areas were clearly defined and compact. Analysis of tissue samples from two additional experiments confirmed that GSC23 control cells developed tumors with irregular borders and finger-like projections into the mice brain (Fig. 3C), while GSC23 cells with G⍺12 knockdown formed tumors with smoother and more defined borders. Quantitative analysis of the shape of the tumor border from sections shown in (Fig. 2A) was carried out using digital pathology QuPath software. While only two representative images were used to provide quantitative data, the multiforme tumor-stroma interfaces were significantly decreased in virtually all G⍺12 KD compared to the control tumors, indicative of diminished invasiveness (Fig. 3B). To further verify these histological observations, we visualized the GSC cells in the tumor by IHC, staining for a human nucleolar antigen protein; this further revealed micrometastasis along the tumor borders in control but not in KD tumors (Fig. 3C). We also demonstrated that G⍺12 knockdown in HK281 cells diminished the invasiveness of tumors formed in vivo (Fig. 3D). These data support the hypothesis that GPCR ligands in the tumor microenvironment utilize G⍺12 to trigger GSC invasion.

### RNA-seq analysis of differentially expressed genes in G⍺12 knockdown and control GSC23 tumors

**Figure 4:**
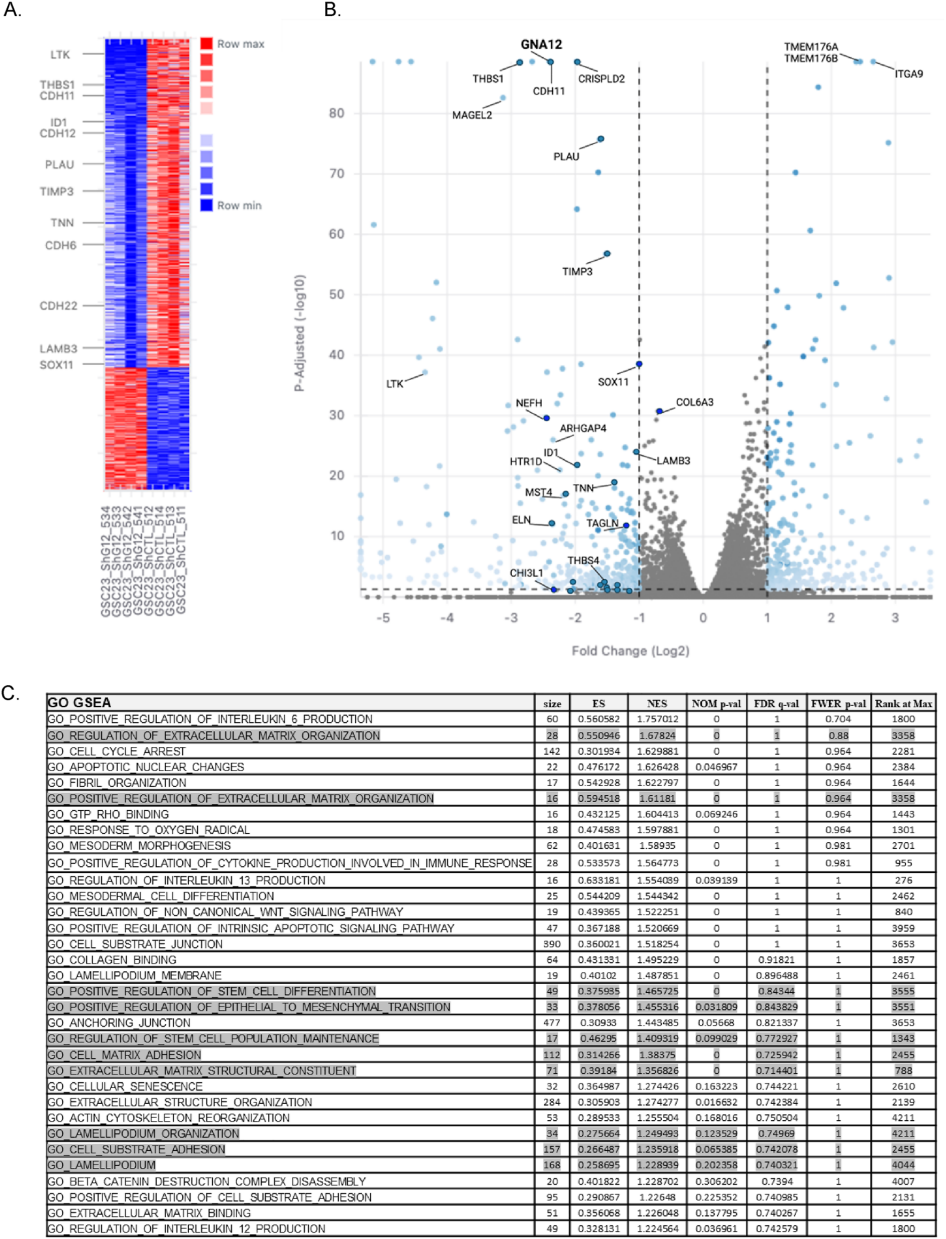
Differentially expressed genes in G⍺12 knockdown GSC23 tumor. (**A**) Heatmap obtained for DESeq2 analysis of GSC23 tumors (shG⍺12#3 or shControl) obtained at 17 days post injection (n = 4, *p-adj* = 0.05). (**B**) Volcano plot shows genes down or upregulated in shG⍺12 KD GSC23 tumor samples. (**C**) Gene Ontology (GO) annotation based on Gene-set enrichment analysis (GSEA) performed to assess biological function and related hallmark pathways for differentially expressed genes in control and G⍺12KD tumors. Those most relevant to tumor phenotype described here are highlighted.

To explore molecular pathways downstream of GNA12 we performed a comprehensive analysis of gene expression profiles in tumors derived from control and G⍺12-depleted GSCs. We harvested tumors at 21 days and submitted 8 GSC23 tumor samples (4 WT and 4 G⍺12 knockdown) for RNA sequencing (RNA-seq). Data generated from the RNA-seq analysis identified 22,247 expressed genes with high confidence. Of these 272 genes were upregulated and 558 were downregulated in G⍺12-depleted tumors (*p*-adjusted < 0.05) as shown in the Heatmap and Volcano plot (Fig. 4A). A more stringent cut-off value of *p-*adjusted < 0.01 was used to rank the most significantly differentially regulated genes annotated in the Volcano plot. Gene oncology (GO) and Gene Set Enrichment Analysis (GSEA) were used to assess the pathways that were influenced by G⍺12 (Fig. 4B).

Genes involved in the regulation of ECM components and organization, matrix adhesion and lamellipodia dynamics as well as stem cell properties and epithelial mesenchymal transition were differentially expressed as shown by RNA-Seq (Fig. 4B). The list includes several markers indicative of a decreased mesenchymal phenotype, for example expression of *CHI3L1* (encoding *YKL-40)* was downregulated over fivefold in 3 of 4 G⍺12 KD tumor samples. *THBS1*, which encodes a matricellular protein and has been proposed as a robust clinical marker of the mesenchymal phenotype and GBM prognosis^25,26^, was downregulated by 80% in the G⍺12 KD tumors. Cadherin-11, a cell–cell adhesion molecule that is associated with EMT/PMT^27^, was also significantly downregulated in G⍺12 KD tumors; this gene is also highly expressed in GBM patient samples in TCGA data analysis (Supplemental Fig. 5). The categorization includes many genes that are in several GO categories since the biological process of EMT, stemness and cell migration are interrelated.

**Figure 5:**
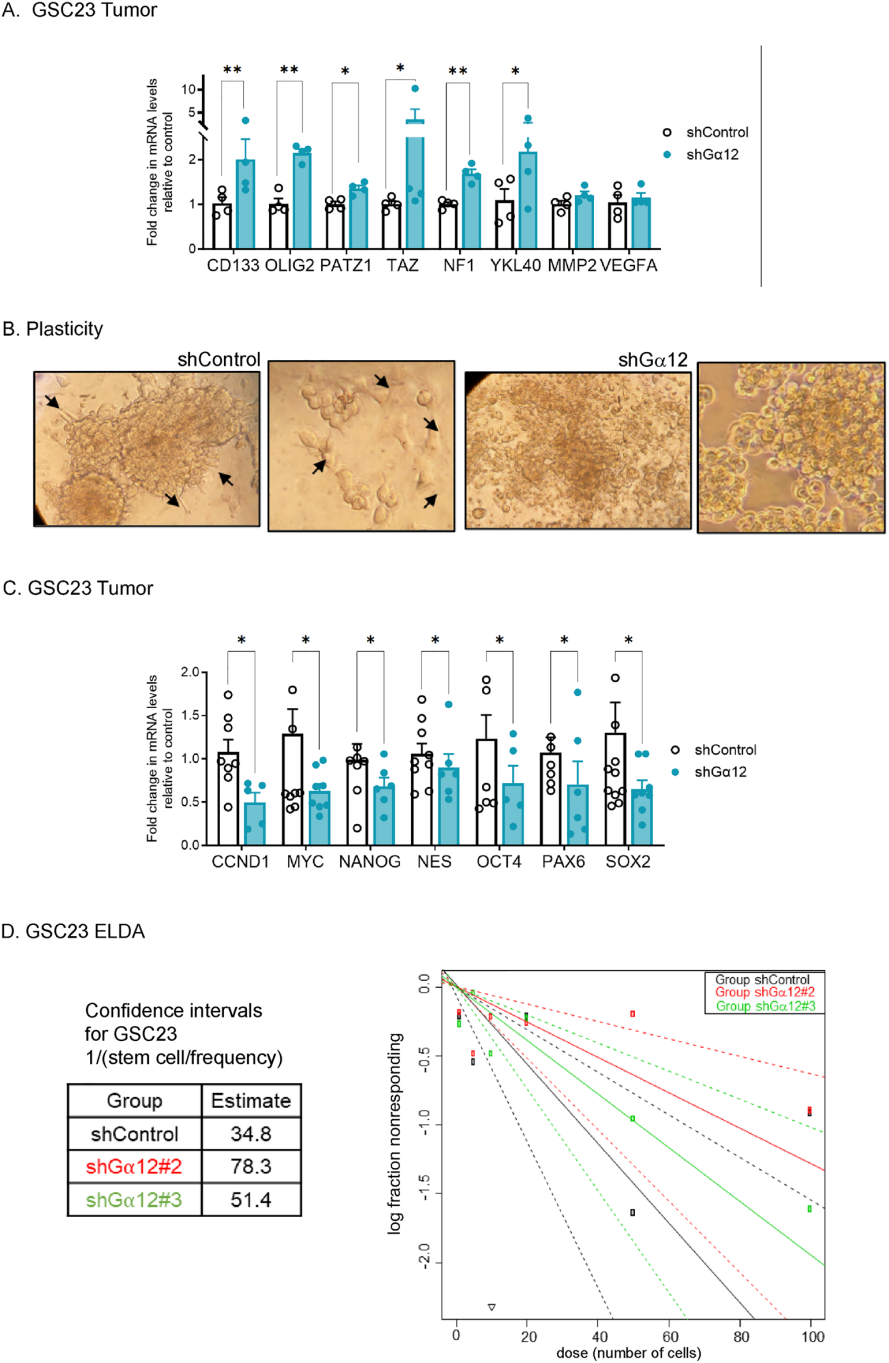
G⍺12 knockdown GSCs tumors have altered gene expression profiles compatible with phenotypic changes in GSC cell behavior observed in vitro. (**A**) mRNA expression of proneural-mesenchymal transition related genes in GSC23 tumor samples (n = 4, *p < 0.05, **p < 0.01 vs. shControl). (**B**) Cell plasticity of GSC23 grown on Matrigel-coated surfaces for 21 days visualized by light microscopy (20X). (**C**) mRNA expression of cancer-associated stem cell genes in GSC23 tumor samples (n = 8, *p < 0.05 vs. shControl). The total number of GSC23 spheres formed at 14 days in culture plotted by extreme limiting dilution analysis (ELDA, 0.95 confidence interval). Estimated stem cell frequency per group in the table.

A proneural-to-mesenchymal transition (PMT), similar to EMT, has been described for GBM^24,28^*.* To more specifically assess genes associated with the proneural/mesenchymal transition (PMT) characteristic of glioblastoma we looked for changes in known PMT associated genes in the GSC23 tumors by qPCR (Fig. 5A).

We observed increases in well-known proneural genes in G⍺12 KD tumors (Fig. 5A), specifically up-regulation of *CD133*, *OLIG2*, and *TAZ* along with a trend towards an increase in *PATZ*. The *NF1* gene, disputably proneural^29^, was also increased as was *YKL40* which was*,* however, significantly down-regulated by RNA-Seq. Overall, these data are consistent with deletion of G⍺12 reducing in vivo proneural to mesenchymal transition and leading to attenuated mesenchymal tumor cell properties. We also demonstrated in an in vitro analysis that the typical plasticity of cancer stem cells seen with prolonged culture (21 days) on Matrigel-coated plates was altered by G⍺12 knockdown (Fig. 5B). Control GSC23 cells formed large and stable spheres, with adherent cells migrating out of the spheres, while G⍺12 KD cells formed less stable spheres that yielded loose rounded-shape cells, consistent with an altered mesenchymal/proneural dynamic state.

We also analyzed mRNA levels of core cancer-associated stem cell genes in G⍺12 KD GSC23 (Fig. 5C) and in HK281 tumors (Supplemental Fig. 4B,C). We observed reduced mRNA levels for seven stem cell genes (*CCND1*, *MYC, NANOG*, *NESTIN*, *OCT4*, *PAX6* and *SOX2*) in the G12 KD GSC23 tumors. To demonstrate that there were functional differences associated with these genetic changes we analyzed GSC self-renewal by in vitro sphere formation comparing control and two lentiviral constructs of G⍺12shRNA knockdown cells (Fig. 5D). Decreasing G⍺12 expression in GSC23 cells lead to diminished stem cell frequency assessed by extreme limiting dilution (ELDA) analysis. GSC23 control cells showed an average of one stem cell for every 35 cells, while the two shRNA knockdown cells averaged one for every ~ 50–80 cells, i.e., the knockdown of G⍺12 protein decreased the ability to generate new spheres by an average of 60% (Fig. 5D). Diminishing G⍺12 mRNA levels in HK281 cells, as in the GSC-23 cells, decreased stem cell properties as assessed by alterations in stem cell gene mRNA levels and growth in the extreme limiting dilution assay (Supplemental Fig. 4).

### G⍺12 promotes a mesenchymal-like invasive phenotype through THBS1 signaling

**Figure 6:**
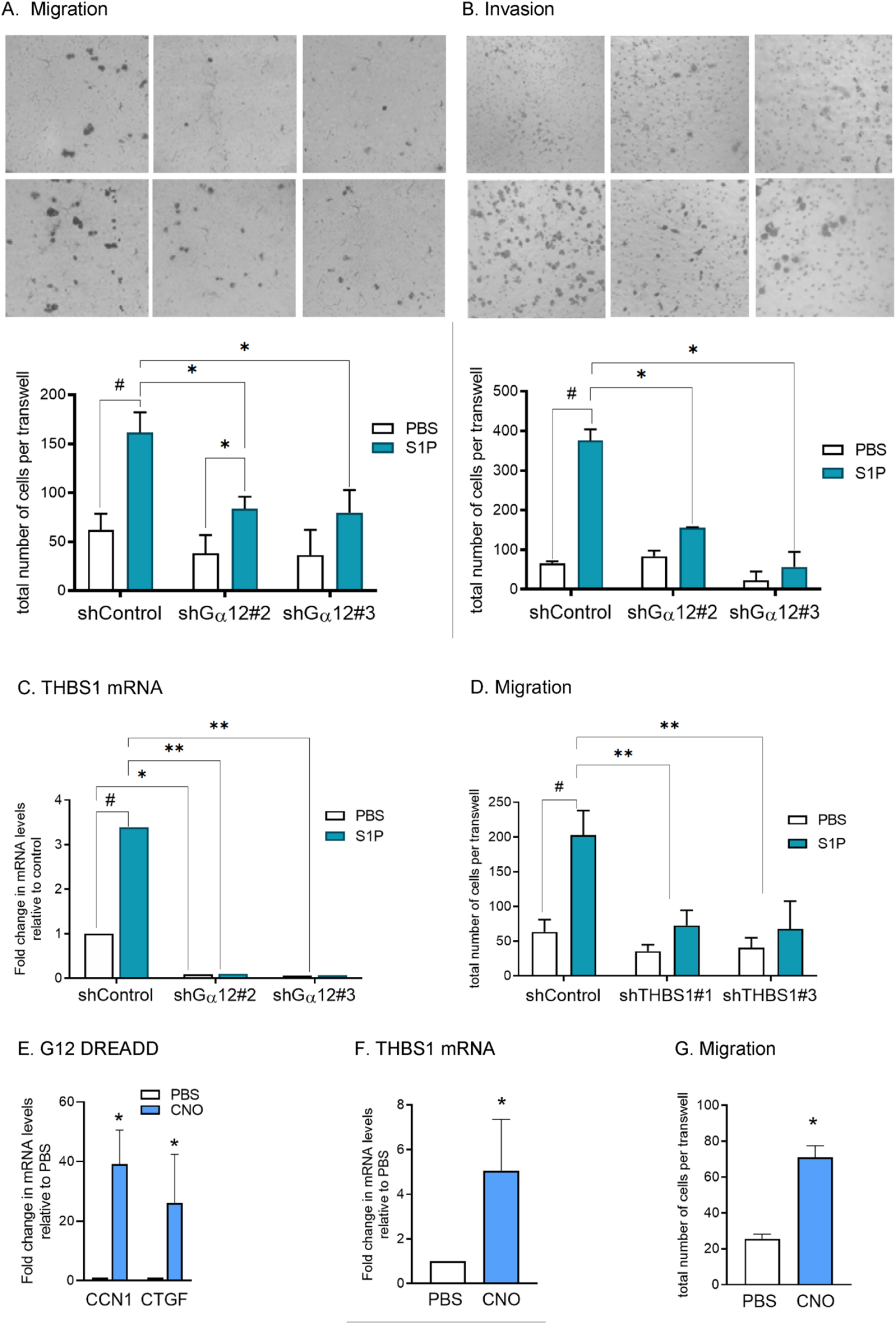
Cell migration and invasion of GSC23 cells are dependent on G⍺12 and Thrombospondin-1. Migration (**A**) or Invasion (**B**) of control or G⍺12KD GSC23 cells through microwells in response to 16 h treatment with 1.0 µM S1P is mediated through G⍺12 (n = 3, *p < 0.05). (**C**) Thrombospondin-1 gene expression (*THBS1*) in GSC23 cells is stimulated by S1P and mediated through G⍺12. (n = 3, *p < 0.05, **p < 0.01 vs. shControl). (**D**) Migration elicited by S1P is blocked by knockdown of *THBS1*. (n = 3, ^#^*p* < *0.05 PBS vs S1P*; **p < 0.01 KDs vs. shControl.). (**E**) G⍺12 DREADD expressing GSC23 cells stimulated by 10um of synthetic ligand CNO express canonical RhoA regulated genes CCN1 and CTGF. (**F**) THBS1 mRNA expression is induced by 10um CNO in G⍺12 DREADD expressing GSC23 cells (n = 3, *p < 0.05 vs. shControl). (**G**) Cell migration elicited by G⍺12 DREADD activation of 10um CNO treatment (n = 3, *p < 0.05 vs. PBS).

To further investigate the role of G⍺12 in GBM tumor invasion we examined the effect of GNA12 knockdown on GSC migration and invasion in vitro (Fig. 6A,B). We used sphingosine-1-phosphate (S1P) to activate GPCRs coupled to G⍺12 and effected a 2.5-fold increase in cell migration and a fivefold increase in invasion. Migration and invasion were significantly attenuated by G⍺12 KD supporting the role for G⍺12 signaling in GSC23 cell migration and invasion. There was no significant effect of S1P on proliferation of either control or G12 KD cells over this time period nor throughout 5 consecutive days, as assessed by Cyquant analysis (Supplemental Fig. 4D).

THBS1 was the one of the most highly down regulated genes in the RNA seq analysis and it has been functionally associated with cell migration, EMT and stemness. We confirmed the decrease in *THBS1* expression demonstrated in the RNA-seq data of G⍺12 KD tumors by qPCR on GSC23 tumor samples (Supplemental Fig. 2E). Accordingly, we focused on THBS1 to interrogate the role that downstream transcriptionally regulated targets of G⍺12 play in GSC23 migration. S1P treatment of GSC23 cells increased *THBS1* mRNA and this increase was fully abrogated by G⍺12 KD (Fig. 6C). Notably basal levels of *THBS1* were also downregulated in the G⍺12-knockdown cells. To investigate the role of THBS1 in GSC23 cell migration we generated THBS1 KD GSC23 cells using lentiviral shRNA (Supplemental Fig. 2E). Migration stimulated by S1P was attenuated by nearly 80% in THBS1-depleted GSC23 cells (Fig. 6D). A gain of function approach was then used to further establish that pharmacological and specific activation of G⍺12 can regulate THBS1 expression and cell migration. GSC23 cells were engineered to express a G⍺12-coupled designer receptor (DREADD)^9^. Activation of the DREADD-expressing GSC23 cells with CNO (the synthetic ligand for the DREADD) was confirmed to be effective based on robust expression of two canonical G⍺12 and RhoA regulated targets genes, *CYR61/CCN1* and *CTGF/CCN2*^7^ (Fig. 6E). We further demonstrated that CNO treatment increased *THBS1* mRNA in GSC23 cells expressing the G⍺12-coupled DREADD (Fig. 6F) and concomitantly increased cell migration (Fig. 6G).

**Figure 7:**
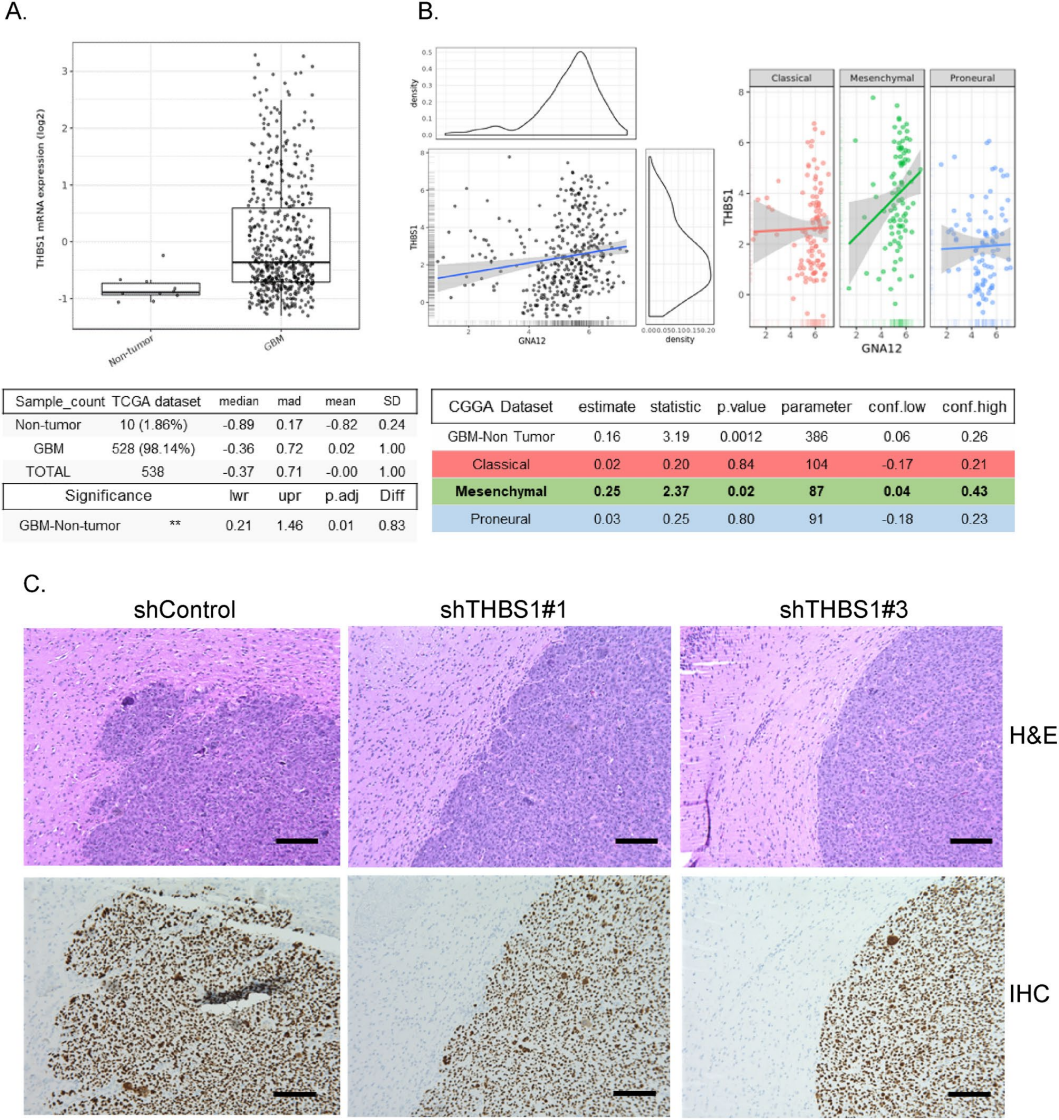
Association of G⍺12 and thrombospondin-1 expression in mesenchymal GBM and requirement of THBS1 for GSC tumor invasion. (**A**) *THBS1* is elevated in GBM patients in TCGA database assessed by GlioVis. (**B**) CCGA dataset indicates positive correlation between *GNA12* and *THBS1* in GBM patient tumor samples classified as mesenchymal. Pearson’s correlation, HSD *p* < 0.01. (**C**) Mouse brain cross sections showing the effect of shTHBS1#1 and shTHBS1#3 KD compared with shControl at 17 days post intracranial injection (H&E or IHC for human nuclei). (HPM, × 40).

Analysis of TCGA confirmed that *THBS1* is highly upregulated in GBM (Fig. 7A). Notably *THBS1* mRNA levels also correlated with those of G⍺12 for mesenchymal tumors in TCGA and the more extensive Chinese Glioma Gene Atlas (CGGA) (Fig. 7B). To evaluate involvement of *THBS1* in GBM growth in vivo we implanted mice with GSC23 cells in which either of two shRNA constructs were used to knockdown *THBS1*. (shTHBS1#1 and #3; Supplemental Fig. 2). There were no significant differences in survival or onset of neurologic sequealae compared to controls after 3 weeks mirroring the lack of effect of G⍺12 knockdown on in vivo tumor growth of GSC23. However, brain sections of mice bearing GSC23 THBS1 KD cells revealed tumors that were less invasive (Fig. 7C). Taken together, these data indicate that *THBS1* is a G⍺12-regulated gene critical for cell migration and GBM invasion.

## Discussion

In this study, we demonstrate a unique and critical role for the heterotrimeric G-protein, Gα12, in GBM. Signaling through heterotrimeric G-proteins depends on their activation by GPCRs, thus our findings implicate endogenous GPCRs and their locally generated ligands in driving GBM tumor progression. G-protein coupled receptors are upregulated and implicated in growth and invasion of numerous cancer types^15,18^. Recently more than 30 GPCRs were demonstrated to couple to Gα12/13^9^ and, based on their downstream signaling, are likely to regulate cancer progression. We established that many of these receptors had altered profiles in GBM samples included in the TCGA PanCancer dataset, including upregulation of *S1PR2, LPAR4, EDNRA, FFAR4, HTR7*, and *OXGR1*. Most strikingly, however, *GNA12* was altered in almost one third of the profiled samples, a higher rate than that observed for any of the Gα12-coupled GPCRs. *GNA12* was notably upregulated in tumor samples largely distinct from those showing overexpression of these GPCRs. Accordingly, even tumors in which GPCRs are not upregulated would be stimulated through Gα12 regulated pathways in a microenvironment in which their ligands (e.g., thrombin, S1P and LPA) are generated.

Knockdown of G⍺12 decreased G⍺12 mRNA without compensatory increases in G⍺13 mRNA, and with an associated decrease in G⍺12 protein. Tumors observed 2-to-3 weeks after implantation of G⍺12KD GSC23 or HK281 cells did not differ in size nor was there a difference in survival of tumor-bearing mice compared to WT controls. The lack of effect on tumor size likely results from the presence of multiple potential stimuli in the tumor microenvironment that could act independently of G⍺12-coupled receptors to stimulate tumor cell growth^30^, and indeed there are multiple pathways for YAP activation and YAP-mediated cell proliferation that would remain intact in the KD cells^31,32^. Consistent with these findings knockdown of G⍺12 did not alter in vitro GSC23 cell proliferation; in addition G⍺12 or G⍺13 deletion has also been reported to have no effect on in vivo growth of pancreatic, breast, or oral cancer cell-derived tumors^21,33,34^.

On the other hand, tumor cell invasion appears to be highly dependent on G⍺12, as it was significantly diminished in the G⍺12 KD tumors, demonstrated in three separate experiments. This was shown in experiments using both GSC23 and HK281 cells, suggesting that it is a generalizable feature of G⍺12 signaling. Our in vitro experiments confirmed this observation, demonstrating that GSC23 cell migration and invasion were enhanced by S1P in control cells, but not in cells in which G⍺12 was knocked down. Our G⍺12 knockdown studies were complemented by gain-of-function experiments using GSC23 cells expressing a DREADD coupled to G⍺12, in which we demonstrated ligand induced activation of cell migration. Defects in cell migration and invasion were also seen in pancreatic and breast cancer cell derived-tumors in which G⍺12 and or G⍺13 were deleted^33,34^, a finding extended by our in vivo orthotopic observations.

G⍺12 and G⍺13 couple to RhoGEFs thus their primary effect is the activation of RhoA. Actin cytoskeletal changes could acutely alter cell shape, motility and migration, well established responses induced through RhoA signaling^3,35^. It is now evident, however, that RhoA activation also leads to transcriptional responses.^6,7,11,13,20^. Our findings with Gα12 KD glioma stem cells suggest that signaling through Gα12 induces transcriptional responses including genes that characterize mesenchymal-like and stem cell-like states and which would contribute to their invasive phenotype. Of related interest, studies examining DNA copy number alterations in GBM using large-scale network modeling identified *GNA12* as a major hub correlated with disease-relevant transcriptional effects^36^. Together these findings support the hypothesis that G⍺12 activity regulates tumor cell migration and invasion through chronic and sustained transcriptional alterations.

A proneural-to-mesenchymal transition (PMT), has been described for GBM^24,28^. This resembles the epithelial-to-mesenchymal transition (EMT) observed in other solid cancers, which has been associated with increased stemness and metastasis^37^. Suggestive evidence for a role of G12 signaling pathway in EMT has been reviewed^38^, but transcriptional responses mediated through G⍺_12/13_ signaling have not been previously linked to the proneural-mesenchymal transition (PMT) in glioma cells. Notably, however, glioma stem cells in early passage culture tend to revert to a less aggressive phenotype with a different molecular signature than that of their parental GBM^39^, and the addition of serum, which contains activators of GPCRs coupled to G⍺12/13 and to RhoA mediated gene expression^6^, stimulates their transition to a more mesenchymal phenotype^24^. In addition, expression of the GBM associated gene RPHN2 (rhophilin) activates RhoA and was also reported to lead to mesenchymal transition of GBM cells^40^. The possibility that transcriptional responses regulated by G⍺12 promote the process of PMT is further supported by our finding that knockdown of G⍺12 in GSCs leads to increases in several genes reflective of a more proneural signature, in particular *OLIG2*, *PATZ1*, and *TAZ*^28,41,42^. In addition, expression of *YKL40* considered by most to be an early marker of a mesenchymal shift in recurrent GBM^43,44^ was reduced.

Our data demonstrate changes in stem cell frequency and expression of canonical stem cell genes, including key transcription factors like NANOG, SOX and NES, were decreased in the G12 knockdown cells. In addition, classical EMT protein families that modulate cell communication processes, e.g. cadherins (CDH11), collagens, and focal adhesion components (integrins) were highly differentially expressed in the tumor samples. Thus while our data do not conclusively support a PMT shift associated with G⍺12 deletion, as defined by GSEA using the Verhaak classification method^19^, or demonstrate all of the phenotypic changes associated with altered stemness, this is not unexpected since GBM presents a high degree of phenotypic variability due to its inter- and intra-tumor heterogeneity. Overall, however, the genomic changes we observed, along with the decrease in the tumor’s aggressive features, are compatible with transcriptional reprogramming through altered expression of proneural-mesenchymal and stem cell genes.

We identified thrombospondin-1 (*THBS1*), associated with the most aggressive and invasive GBM tumors, as one of the most highly downregulated G⍺12 dependent genes in the tumor samples analyzed by DESeq2, demonstrating that *THBS1* expression was decreased by 90% in G⍺12 KD tumors. We extended our analysis using GSCs in vitro, demonstrating that *THBS1* expression was induced through G⍺12 signaling by S1P, as well as by direct activation of G⍺12 through ligand stimulated DREADD activation. Previous work has linked TGFβ/STAT3 signaling to *THBS1* expression^25^, but to our knowledge, the data we present are the first to implicate GPCR and G⍺12 signaling in expression of thrombospondin-1. Notably, our analysis of the promotor of the *THBS1* gene sequence using prediction tools for transcription factors binding sites (e.g., TRANSFAC-based public tools PROMO or MATCH, and TFBSPred, https://www.michalopoulos.net/tfbspred/) revealed a variety of biding sites, including those for MRTFA/SRF and YAP/TAZ/TEADs, transcriptional effectors robustly regulated through G⍺12 signaling.

Our finding that *THBS1* knockdown phenocopies that of G⍺12, with little effect on tumor size but a clear change in invasiveness at the tumor border, suggests that upregulation of *THBS1* through G⍺12 signaling is one of the transcriptional targets that mediate GBM tumor invasiveness. We show here that its expression also correlated with that of G⍺12 in TCGA tumor samples. While the role of THBS-1 as regulator of cell invasion is less well established than its role in angiogenesis^26^, it has been shown to regulate the tumor microenvironment, bind to integrins, and activate several protein kinase pathways involved in cell migration including ERK, p38MAPK, and FAK^25,45,46^. Interestingly, our groups have shown that FAK is activated through integrin signaling downstream of RhoA and ligand induced G⍺12 activation^47^ and through RhoA signaling in uveal melanoma^48^. Thus, it will be of interest to determine if FAK serves as a downstream effector of THBS-1 to mediate invasiveness of GBM.

Taken together, our in vivo and in vitro data suggest that GBM tumor cells respond to endogenous GPCR agonists in the tumor environment to engage G⍺12 transcriptional signaling that alters molecular programming of GSCs. Our findings support the notion that activation of G⍺12 signaling contributes to a phenotypic shift towards a more invasive and mesenchymal tumor growth pattern. Thus, downregulation of this signaling pathway could be efficacious in treating GBM by decreasing therapeutic resistance and the tumor infiltration that contributes to recurrence.

### Supplementary Information

Supplementary Figures.

## Data Availability

RNA-seq datasets have been submitted to the Gene Expression Omnibus (GEO) and the accession number provided is GSE229420.
