# Supplementary material for: Gα12 signaling regulates transcriptional and phenotypic responses that promote glioblastoma tumor invasion
Source: Sci Rep. 2023 Dec 16;13:22412. doi: 10.1038/s41598-023-49164-4 (PMC10725435; doi:10.1038/s41598-023-49164-4)
Supplement: Supplementary file 1 — Supplementary Figures. [file 41598_2023_49164_MOESM1_ESM.docx]

**SUPPLEMENTAL FIGURES**

**
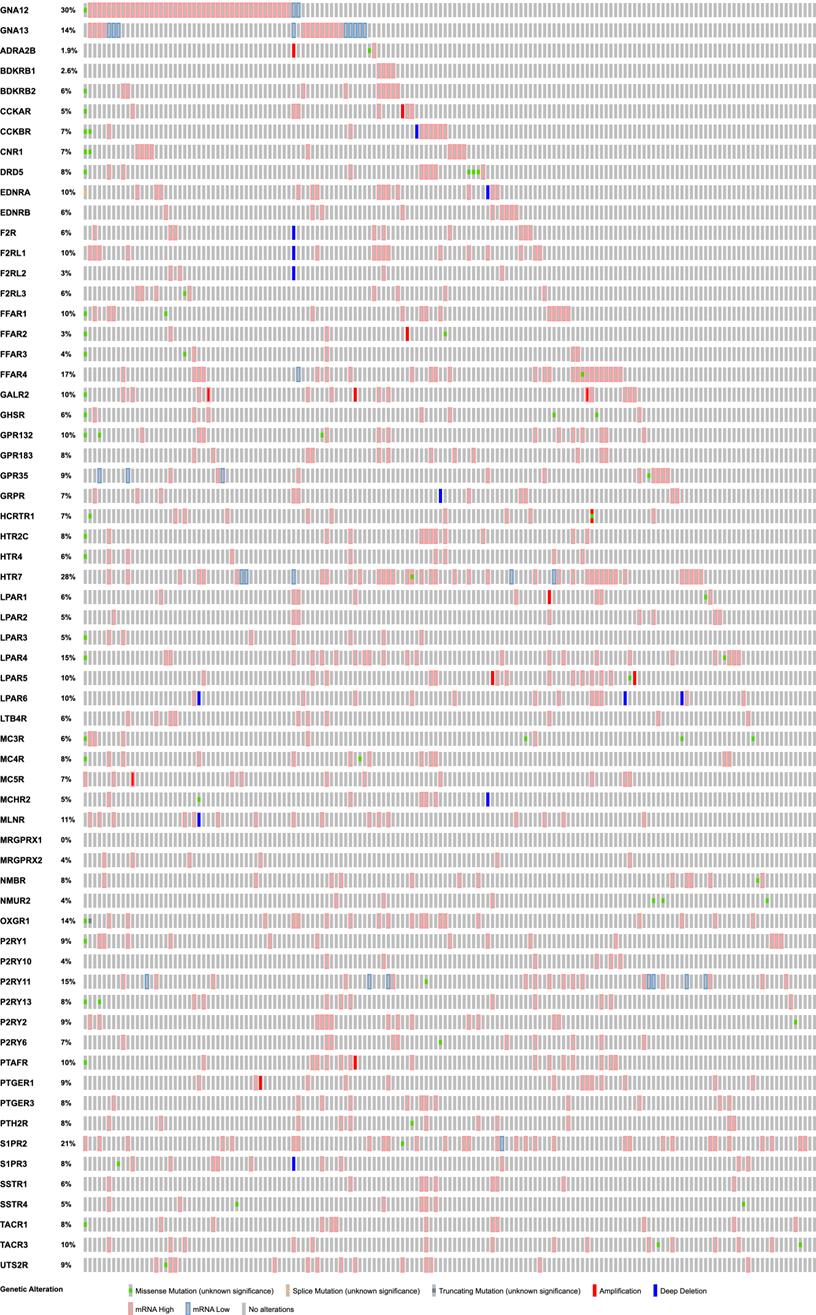
**

Supplemental Figure S1: Extended cBio of G⍺12-GPCRs. Oncoprint for all of the predicted G⍺12-coupled receptors (Inoue *et al.* 2019), based on genomic profiles from Glioblastoma PanCancer Atlas study by cBioPortal (z-scores relative to diploid samples RNA Seq V2 RSEM: +/-1.5 threshold).

B. HK281 tumor

A. GSC23 tumor

GSC23 cells

HK281 cells

Gα12 mRNA

Gα13 mRNA

Gα12 mRNA

Gα13 mRNA

C. GSC23 Gα12 protein


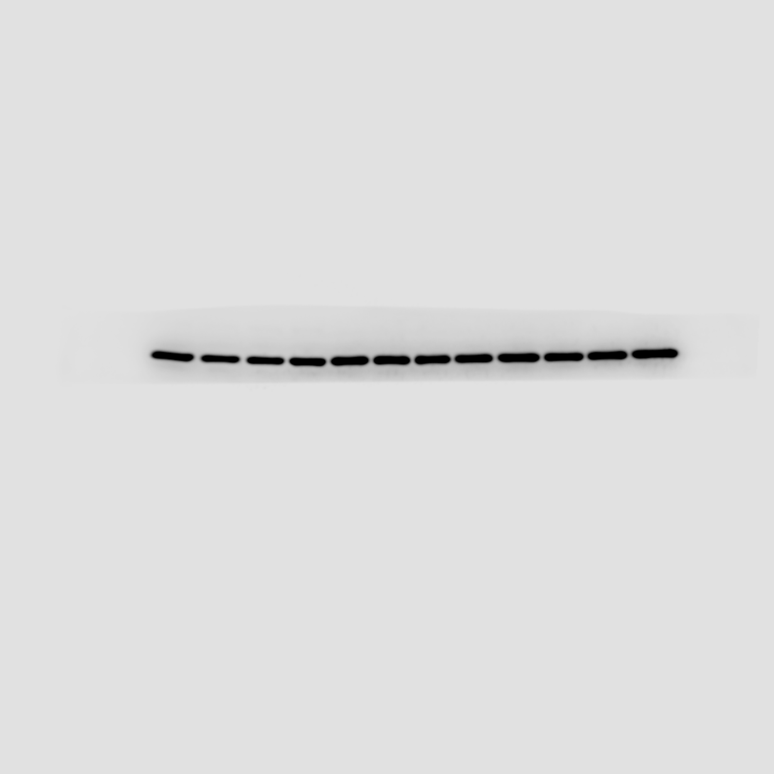


**30**

**RhoGDI**


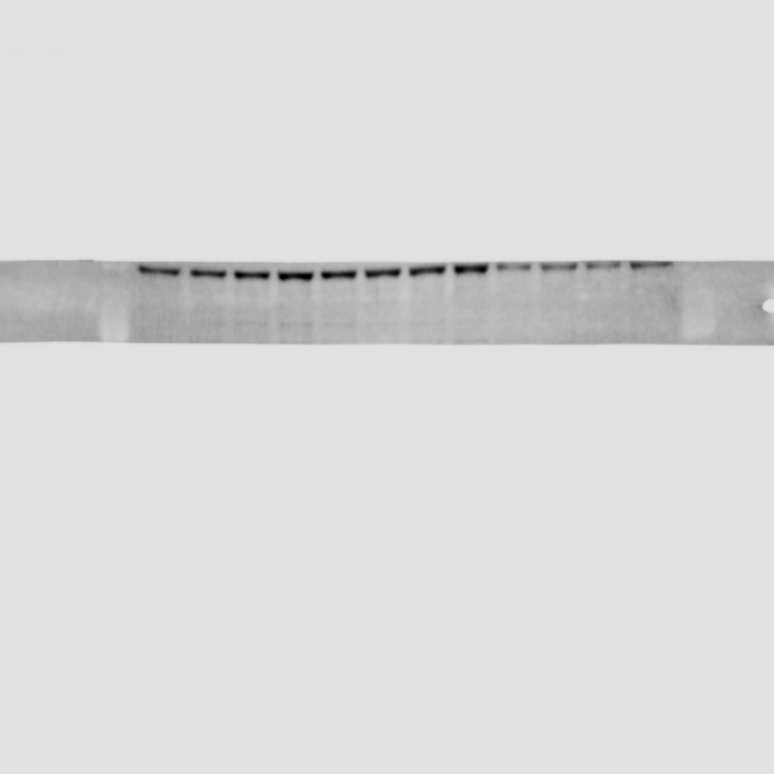


**Gα12**

**50**


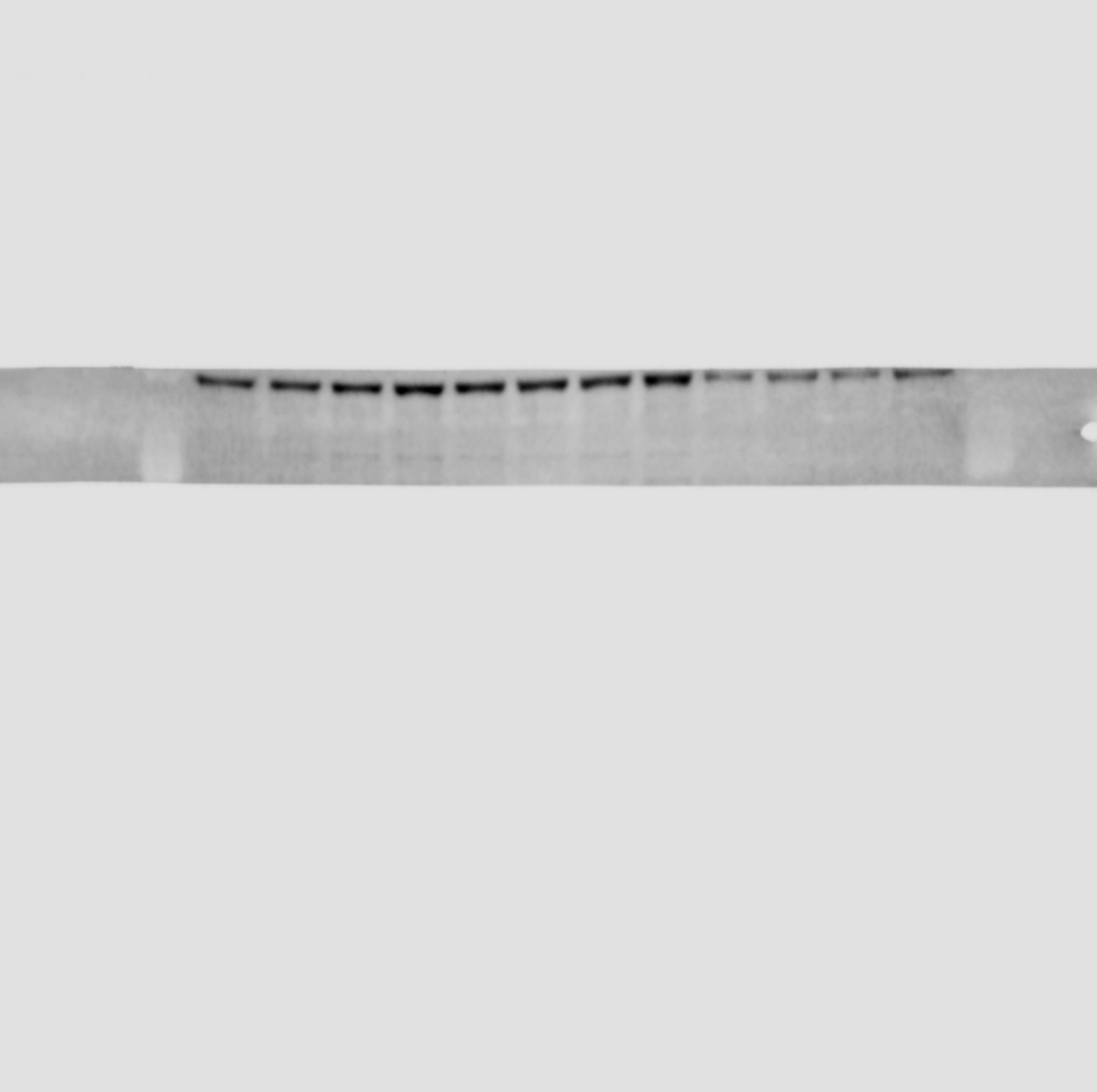

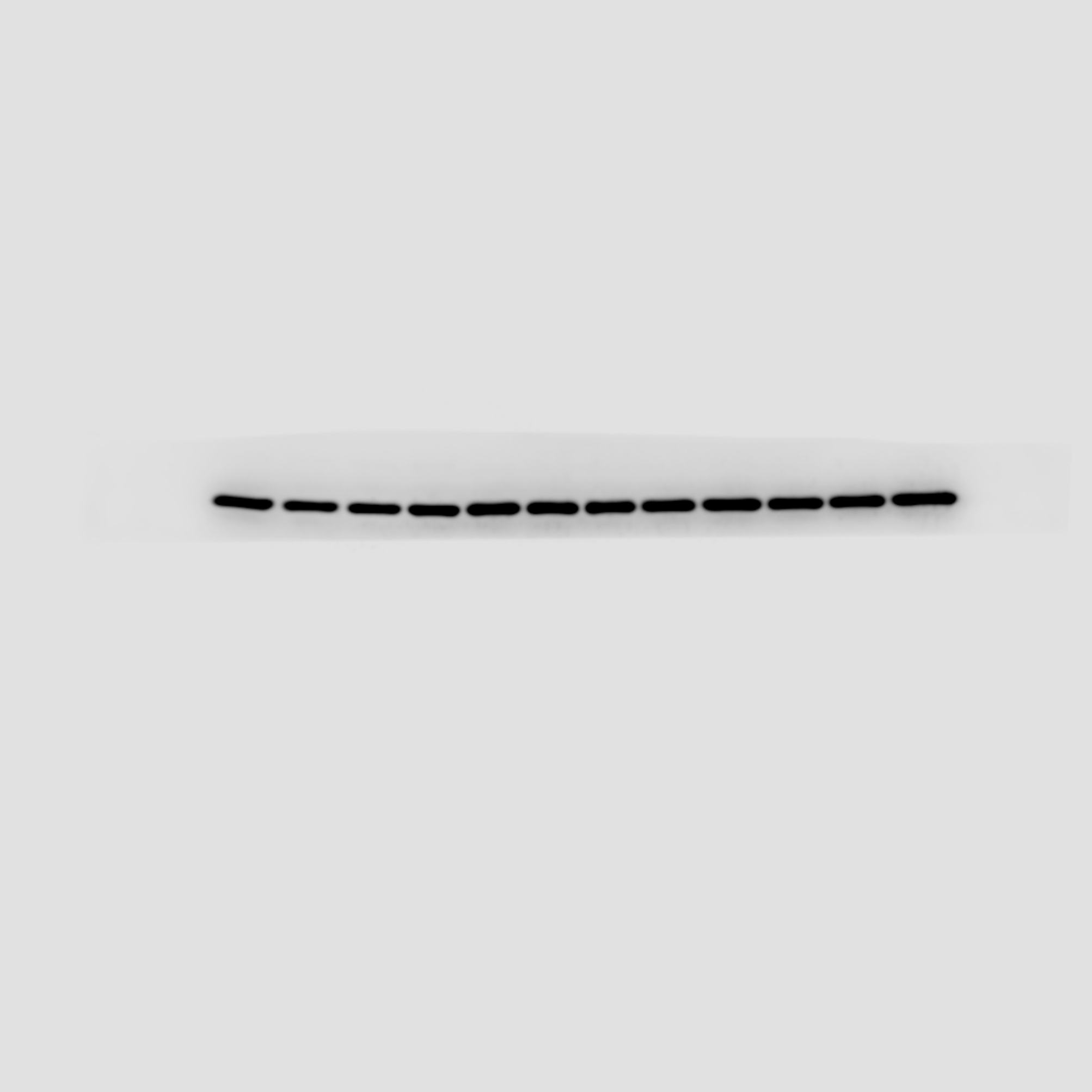


shGα12#3

**RhoGDI**

- 2h 4h 16h

**S1P**

**Gα12**

shGα12#2

- 2h 4h 16h

shControl#1

- 2h 4h 16h

**50-**

**30-**


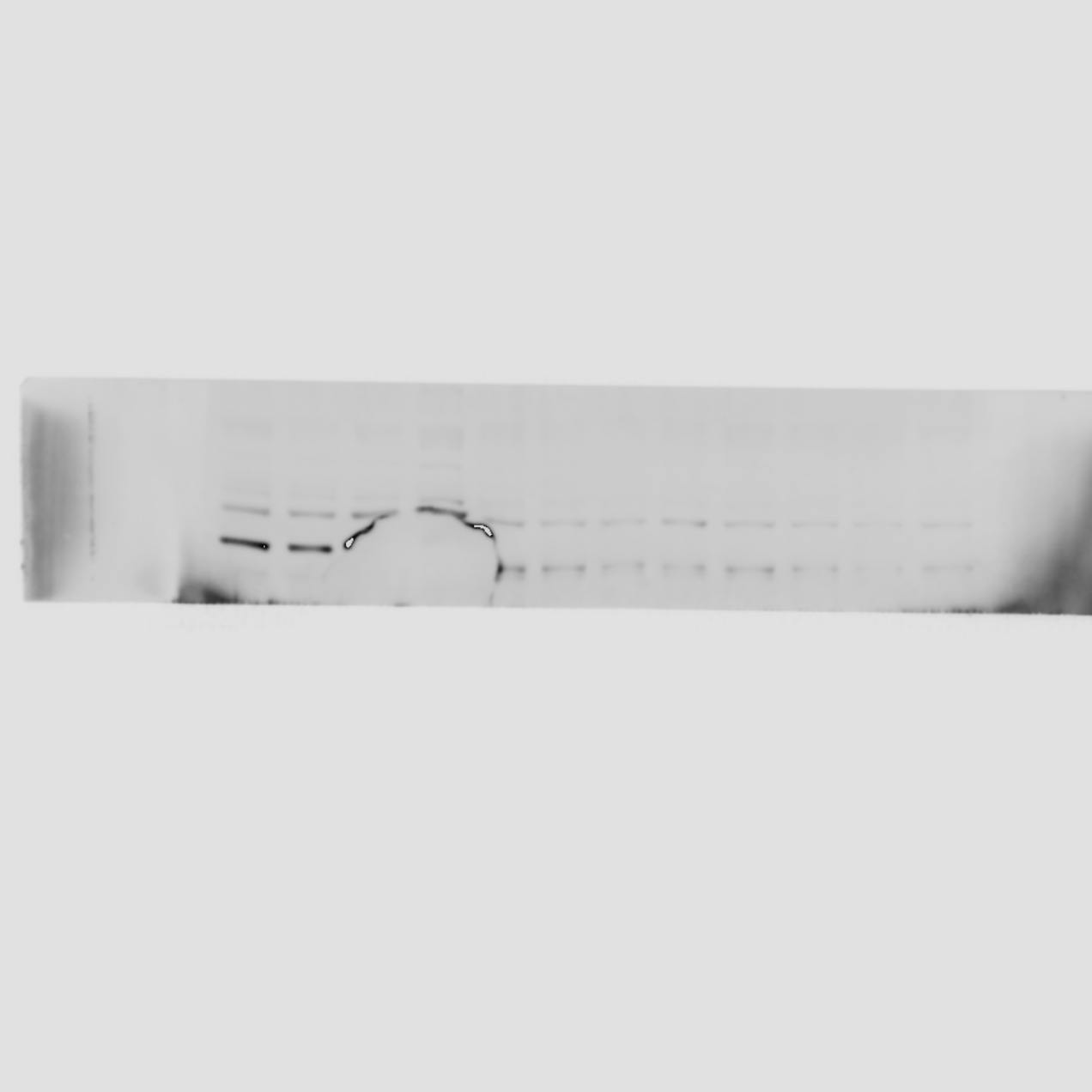


**Gα12** (despite of the bubble issue, a blot performed for shorter S1P time points samples)

**50-**

**37-**

shControl#1

shGα12#2

shGα12#3

E. THBS1 KD cells

D. GSC23 tumor THBS1 mRNA

Supplemental Figure S2: G⍺12 knockdown in GSC-tumor derived samples.

(A-B) Selective G⍺12 knockdown is retained in both GSC23- and HK281-derived tumors.

(n= 4, ***p* < 0.01).

(C) Western blot using anti-G⍺12 polyclonal antibodies showing that shRNA lentiviral knockdown in GSC23 cells reduced G⍺12 protein levels. Incremental pilot for S1P treatment in time courses. Blots were cut prior to hybridization with anti-G⍺12 polyclonal antibodies.

(D) THBS1 mRNA assessed by qPCR is decreased in G⍺12KD derived tumor. (n = 3, *p <0.05 vs. shControl). (E) THBS1 knockdown in GSC23 cells by 3 lentiviral constructs. mRNA expression analysis of THBS1 by qPCR in GSC23 shControl or shTHBS1 knockdown cells (n = 4, *p <0.05, **p < 0.01 vs. shControl). (D) Representative brain sections from G⍺12 KD GSC-23 tumors stained with H&E frequently show necrotic cores (HPM 2x).


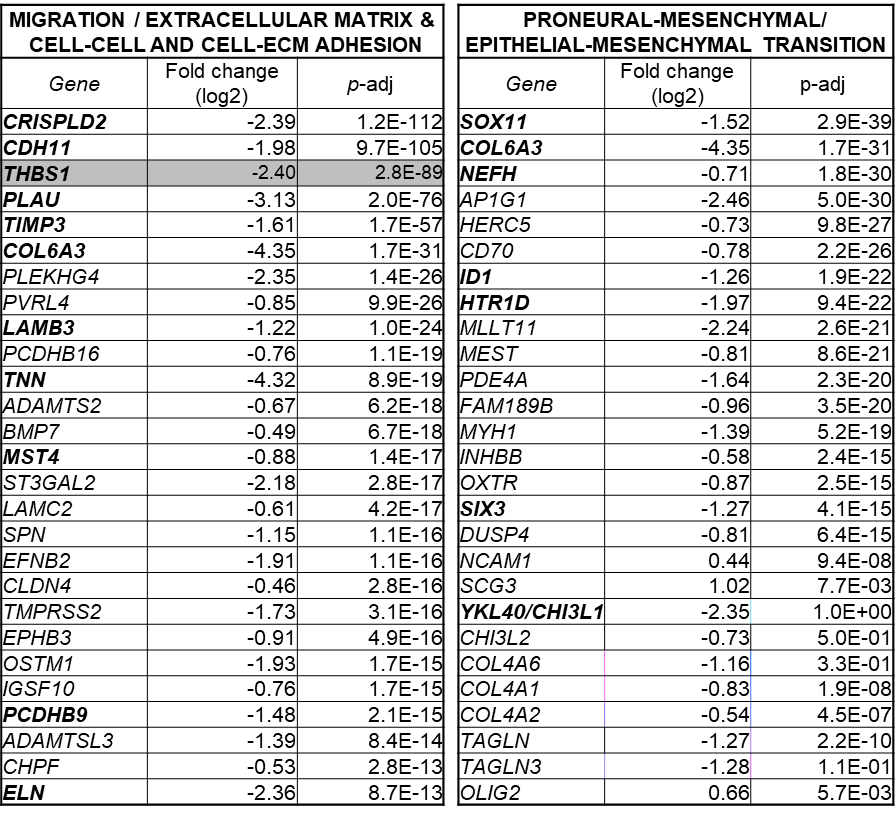


Supplemental Figure S3: DESeq2 analysis reveals G⍺12-dependent genes in GSC23 tumor samples

DESeq2 analysis of GSC23 tumors (4 tumor samples each: shG⍺12, construct #3 or shControl) harvested at 17 days post injection. Genes in bold are labeled in the volcano plot (Figure 5); values in table are expressed as log2fold over shControl tumor samples (*p-adj* < 0.05).


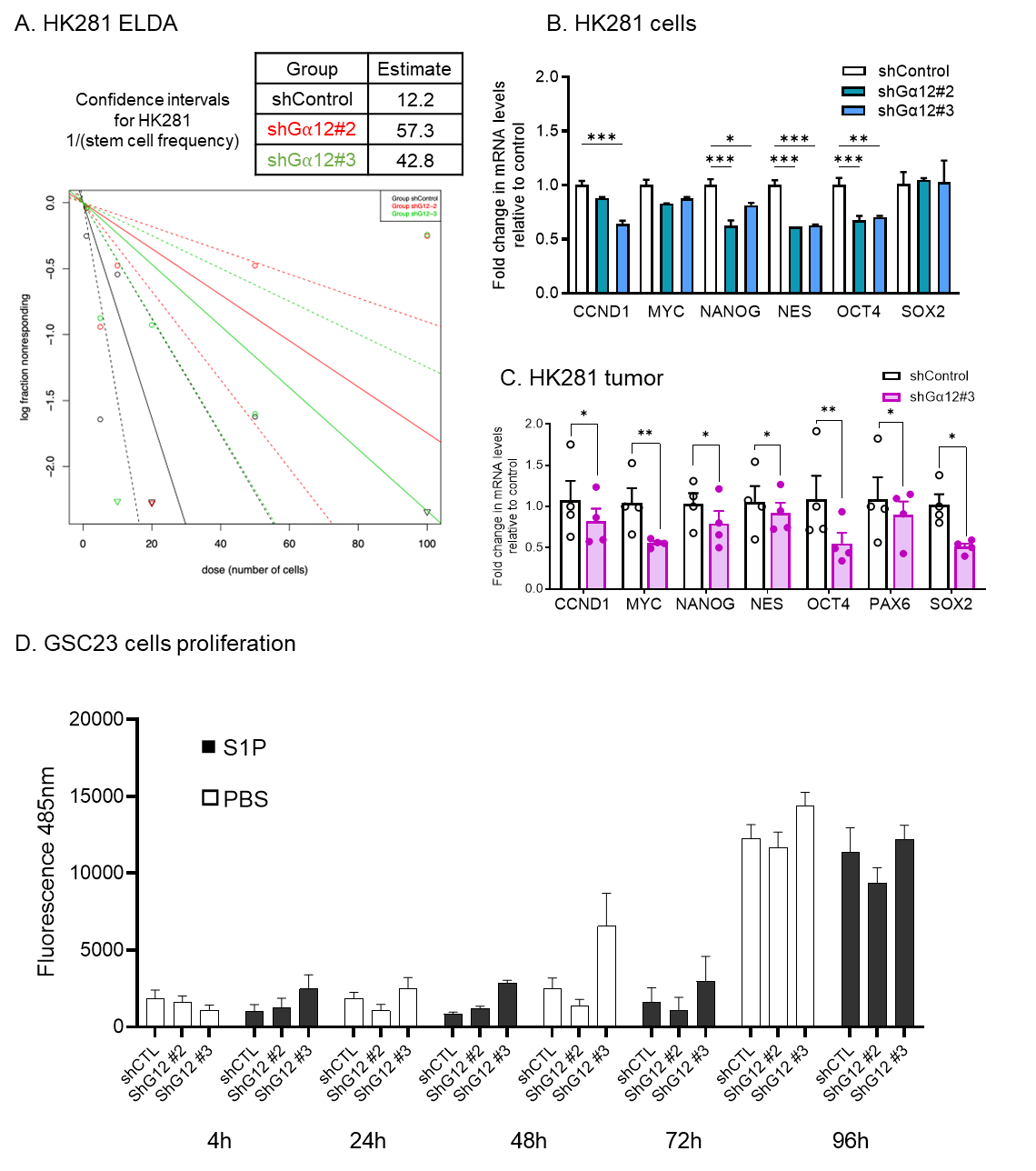


Supplemental Figure S4: Gα12 KD in HK281 cells and derived tumor properties and in GSC23 proliferation. (A) The total number of HK281 spheres formed at 15 days in culture and analyzed by extreme limiting dilution analysis (ELDA, 0.95 confidence interval). Estimated stem cell frequency in each group at the right table. (B-C) mRNA expression of cancer-associated stem cell genes in HK281 cells and tumor-derived samples (n = 4, **p* < 0.05, ***p* < 0.01 vs. shControl). (D) Fluorometric assessment of S1P-stimulated proliferation of GSC23 cells by CYQUANT® NF assay. The experiment was performed in tetraplicates p > 0.05, n.s. vs. shControl).


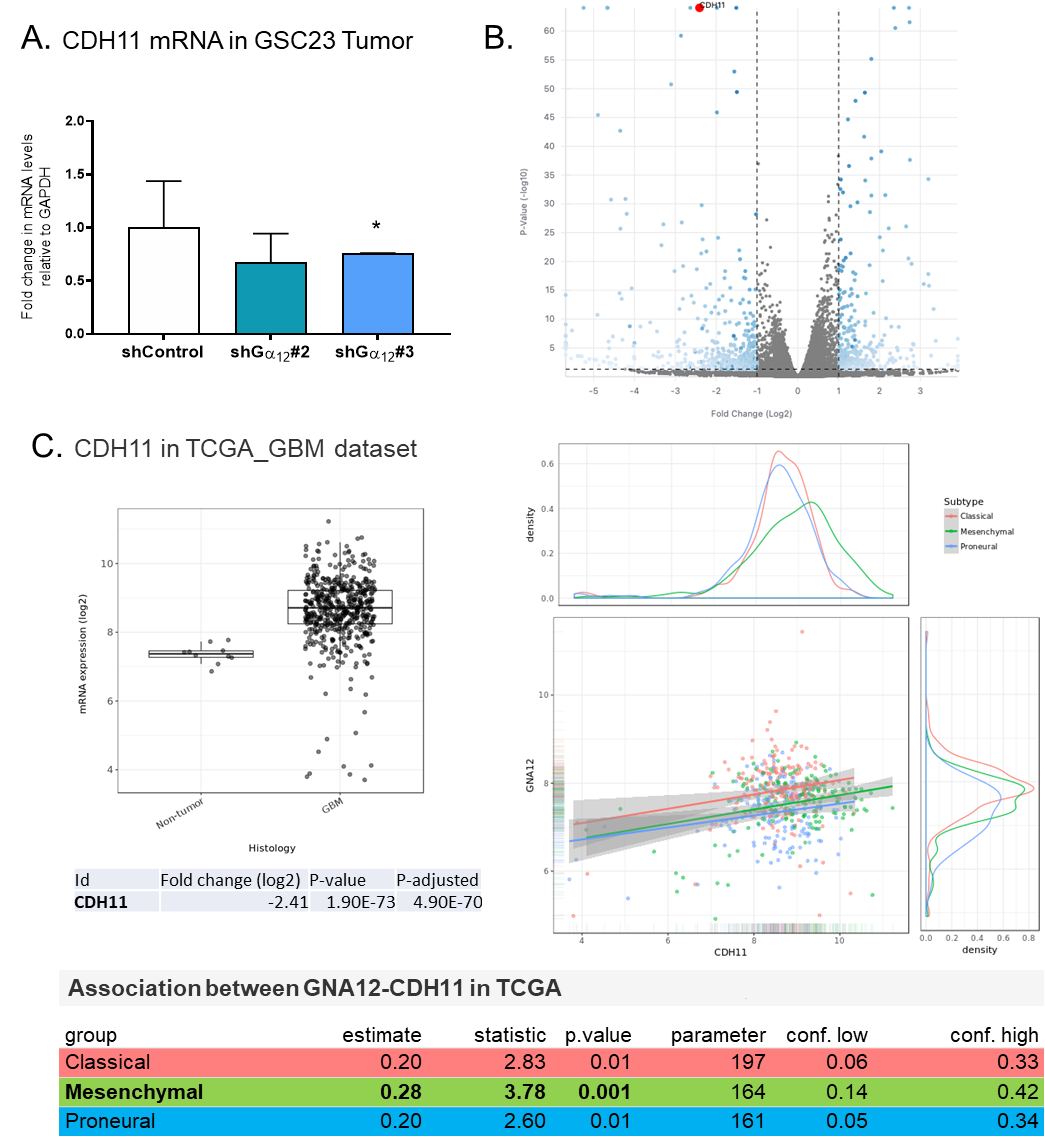


Supplemental Figure S5: Cadherin-11 expression in GSC23 tumor samples and GBM in TCGA database.

(A) Cadherin-11 (*CDH11*) mRNA assessed by qPCR was decreased in Gα12 KD GSC23 derived tumor.

(B) Volcano plot and heatmap visualization of *CDH11* gene in DESeq2 of Gα12 KD tumor vs control.

(C) TCGA dataset show *GNA12* and *CDH11* positive correlation in GBM tumor patients, with higher significance suggested for tumor samples classified as mesenchymal (Pearson’s, HSD ***p*< 0.001) by Gliovis.

**Supplemental Details for Methods**

**Reagents.** Sphingosine-1-Phosphate (S1P d20:1) was from Avanti Polar Lipids (#860662). Growth factor reduced extracellular basement membrane Geltrex was from Gibco (#A1413301). Three non-overlapping shRNA-expressing lentiviral plasmids to human *GNA12* (#1 TRCN0000036755, #2 TRCN0000036756 and #3 TRCN0000036757) were obtained from the La Jolla Institute for Immunology Functional Genomics Core. Human *THBS1* (#1 TRCN0000226402, #2 TCRN0000226405, and #3 TCRN0000226403) and a non-targeting control shRNA (SHC016) were purchased from Sigma-Aldrich. Gα12-specific DREADD was generated and provided by Gutkind lab as hM3D-GPR183/ICL3 receptor with additional 1.57V mutation (3). Human nuclear antigen NM95 antibody for immunohistochemical analysis was from Abcam and Gα12 antibody for western blot was rabbit polyclonal IgG (S-20) from Santa Cruz.

**Lentiviral production.** Lentiviral particles were generated using lentiviral packaging mix (SHP001, Sigma) and aforementioned plasmids in HEK 293T cells. Co-transfection of the packaging/expression vectors used standard PEI method in DMEM/10% FBS. At 16 hours post transfection, medium was changed to fresh neurobasal medium/B27 and cells incubated for 24-48 hours. Virus containing medium was filtered (0.45 μM) and concentrated by ultracentrifugation (Beckman SW-32 rotor,1.5 hours at 26,000 RPM). Lentiviral particles pellets were resuspended in HBSS for immediate use or storage at –80°C.

**Extreme Limiting Dilution Assay.** Neurospheres dissociated into cell suspension by Accutase treatment were counted and plated in fresh media in pentaplicate into 96-wells at 1-100 cells/well. After 21 days spheres were observed using an inverted microscope and counted to estimate stem cell frequency of control vs. knockdown by ELDA, <http://bioinf.wehi.edu.au/software/elda/>.

**Orthotopic GSC Injections.** 1.5 or 5 x10^5^ control or knockdown GSC cells tagged with near infrared IRFP720 were intracranially injected into the mouse brain (6 mice per group), using a stereotactic system as previously described (13). Tumor size was estimated using fluorescence emission detection by FMT 2500 Fluorescence Tomography (Perkin Elmer) at 720 nm. The onset of neurologic sequelae in the control group was used to determine time of euthanasia. Brain samples were collected, and tissue samples were processed for histological examination by H&E and anti-human nuclei IHC at UCSD CALM or MCC Biorepository and Tissue Technology Core. *In vivo* experiments were executed under approval of animal protocol by UCSD IACUC #S00192M.

**RNA Analysis**. Total RNA isolation from GSC neurospheres or tumors was achieved by Trizol reagent and Monarch Total RNA Miniprep Kit (Invitrogen and NEB) according to the manufacturer’s protocol. RNA quantity and 260/280 ratio quality were assessed by Nanodrop. cDNA was obtained from 1ug RNA using a High-Capacity cDNA Reverse Transcription Kit (ThermoFisher). Predesigned primers and Taqman probes were used for mRNA relative quantification by real-time qPCR (Applied Biosystems) using the QuantStudio 3 cycler (Thermo). GAPDH was used for normalization and fold-change calculated by delta-delta Ct method.

**RNA-Sequencing.** RNA tumor samples from mice injected with Gα12 KD and control GSC23 cells (4 mice tumors for biological replicates in each group) were submitted to UCSD IGM Genomics Center for RNA integrity analysis (Agilent Bioanalyzer, Tapestation results eRIN >8.5), ribodepleted library preparation and sequencing using Illumina NovaSeq 6000 (run set PE100 and 25M reads). Raw reads were trimmed, aligned to reference genome hg19 using STAR and differentially expressed genes were calculated using adjusted P-value of <0.05 with a 2-fold change DEseq2 workflow (BasePair software; <https://app.basepairtech.com/>). Gene-set enrichment analysis was performed using GSEA software. RNA-seq data has been submitted to the Gene Expression Omnibus (GEO) and an accession number is GSE229420.

**Migration/Invasion Assay.** Uncoated or Geltrex-coated membranes of Transwell 24-well plates (8µm pore size, Corning) were used for migration and invasion, respectively. GSC single cell suspensions in fresh media (>80% viability post Accutase dissociation) were added into upper chambers and medium plus/minus 0.3µM S1P to the lower chambers. After 16 hours the upper surface of the membrane was swabbed to remove remaining cells and inserts fixed with cold methanol. Cells on the membrane were stained with 0.01% crystal violet, visualized by light microscopy and images taken for counting. Each graph represents two or more independent experiments with duplicates/triplicates of inserts per group.

**GBM Patients Data Analysis.** TCGA and CGGA GBM datasets from GlioVis portal (<http://gliovis.bioinfo.cnio.es>) were used to obtain median cutoff values to compare specific genes expression levels, genomic profiles and other relevant clinical features in histologically characterized samples from GBM patients. Additionally, selected molecular profiled GBMs were visualized at cBioPortal (https://www.cbioportal.org/).

**Statistics.** Comparison between groups for statistical differences were tested using Graphpad Prism software version 8. Analysis of variance (ANOVA) followed by Tukey’s multiple test was applied for groups with several features. One-way ANOVA was used to analyze data from experiments with one independent variable, and two-way ANOVA for two independent factors. Log-rank test was performed to determine Kaplan-Meier survival curves. Data are presented as mean ± SEM and significances based on calculated probability values (**p<* 0.05; ***p<* 0.01). Limiting dilution for stem cell frequency by ELDA used χ2 for pair-wise differences (https://bioinf.wehi.edu.au/software/elda/).
